# Supplementary material for: Vitamin D as a Biomarker of Ill-Health among the Over-50s: A Systematic Review of Cohort Studies
Source: Nutrients. 2019 Oct 6;11(10):2384. doi: 10.3390/nu11102384 (PMC6836048; doi:10.3390/nu11102384)
Supplement: Supplementary file 1 [file nutrients-11-02384-s001.zip › Table S4 Table of results.pdf]

Table S4 Table of results

Afzal 2014

**Biomarker**	**25(OH)D**
**Outcomes**	**Forced expiratory volume in 1s (FEV1)**<br>**Forced vital capacity (FVC)**<br>**Chronic obstructive pulmonary disease (COPD)**
**Categories of vitamin D levels**	Quintiles<br>Reference: the highest quintile
**Results**	*Copenhagen City Heart Study.* Subjects in the lowest seasonally adjusted quintile had a faster decline in lung function in comparison to subjects in the highest, with similar trends in both outcomes (FEV1 and FVC). Results were not significant for never smokers and who quit smoking during follow-up. Risk to COPD in this cohort was significant for the lower two seasonally adjusted quintiles (with OR>1.5), compared to the highest quintile.<br>*Copenhagen General Population Study.* Risk for COPD in this cohort was statistically significant only for the lowest seasonally adjusted quintile in comparison to the highest one (with OR>1.5).
**Conclusion**	There was association of low levels of plasma 25-OHD with a faster lung function decline and with major risk of COPD in both cohorts. No data recorded about vitamin D intake at baseline and during the follow-up period.

Afzal 2013

**Biomarker**	**25(OH)D**
**Outcomes**	**Melanoma skin cancer (MSC)**<br>**Non-melanoma skin cancer (NMSC)**
**Categories of vitamin D levels**	Clinical categories: <25 noml/L; 25-49.99 nmol/L; ≥50 nmol/L; seasonally adjusted tertiles<br>Reference: <25 noml/L in clinical categories and the lowest tertile in seasonally adjusted categories
**Results**	**Clinical categories.** There was not statistically significant association between 25-OHD levels and MSC, whereas the two upper categories were positively associated to NMSC, both for age-and sex adjusted analysis and multivariable adjusted analysis (for gender, pack-years, body mass index, income, occupational physical exertion, intensity of leisure-time activities, and regular cycling or running).<br>**Seasonally adjusted tertiles.** Associations were positive between 25-OHD and NMSC, in both models; there were no differences for MSC: both categories were similarly associated to this outcome.
**Conclusion**	Increasing levels of plasma 25-OHD were prospectively associated to a major risk for MSC and NMSC. Limitations were related to sun exposure and skin phenotype were not recorded, and the minor exposition of the Danish population respect other population.

Al-khalidi 2019

**Biomarker**	**25(OH)D**
**Outcomes**	**Cardiometabolic mortality** (for heart diseases, malignant neoplasm, chronic lower respiratory diseases, accidents, cerebrovascular diseases, Alzheimer's disease, diabetes mellitus, influenza and pneumonia, nephritis, nephrotic syndrome and nephrosis, and all other causes)
**Categories of vitamin D levels**	Quartiles: <30 nmol/L, 30<50 nmol/L, 50<75 nmol/L, ≥75 nmol/L<br>Reference: n.a.
**Results**	Lifetime risks (cumulative risk) to 70 years of age for mortality was highest for <30 nmol/L group and lowest for 50<75 nmol/L group. Above 30 nmol/L lifetime risk was similar for all three groups. Lifetime risks increased with age. Similar trends were found for stratification by BMI status, with less lifetime risk in <30 nmol/L groups independently by BMI. In the overweight group, lifetime risks were similar in all vitamin D categories to 80 years of age and older; in obesity group lifetime risks were similar until 70 years of age independently vitamin D categories. Considering estimates stratified by aggregate risk factor (age, sex, smoking status, systolic and diastolic blood pressure, cholesterol, blood glucose) and BMI, the lifetime risks were lowest for ≥30 nmol/L - low-intermediate risk subgroup in all sample and across BMI categories; the lifetime risks was highest in <30 nmol/L - high risk in overall sample and across all BMI categories except for overweight in which differences related to vitamin D status in high risk subgroups were observed only in 60-80 years aged population.
**Conclusion**	Authors conclude that total vitamin D <30 nmol/L is predictive of high lifetime risk of cardio metabolic mortality, independently by BMI status (but not by risk factor levels).

Arabi 2012

**Biomarker**	**25(OH)D**
**Outcomes**	**Lumbar spine Bone Mineral Density (BMD)**<br>**Total hip Bone Mineral Density (BMD)**<br>**Femoral neck Bone Mineral Density (BMD)**<br>**Trochanter Bone Mineral Density (BMD)**<br>**Forearm Bone Mineral Density (BMD)**
**Categories of vitamin D levels**	25(OH)D levels used as continuous variable
**Results**	Pearson's coefficients of correlations between the percent changes in serum 25-OHD concentrations and BMD reported significative positive associations for trochanter BMD in overall population (0.19, p<0.01), and for total hip BMD (0.31, p<0.05), femoral neck BMD (0.36, p<0.01), and trochanter BMD (0.26, p<0.05) in males. There were non-significative associations for the other outcomes and in female group. There was no difference among subjects with low 25-OHD levels and normal parathyroid hormone (PHT) and who had high 25-OHD levels and normal PHT. Bone loss rate is higher in subjects with high levels of PHT, independently by 25-OHD levels.
**Conclusion**	PTH levels, rather than 25-OHD, predict rates of bone loss in the elderly. Limits were the small sample and the lack of control for sex steroid hormones (as factor for bone loss and fractures risk in elderly). Other limit was the low mean level of 25-OHD in population at baseline to observe associations between serum 25-OHD and bone loss. Finally, the high rate of lost at follow-up could be have affected results.

Aregbesola 2013

**Biomarker**	**25(OH)D**
**Outcomes**	**Pneumonia**
**Categories of vitamin D levels**	Tertiles (8.9- 33.9 nmol/L; 33.9-50.7 nmol/L; 50.8-112.8 nmol/L)<br>Reference: 50.8-112.8 nmol/L
**Results**	During the 9.8 years of follow-up, subjects in the lower tertile had HR of 2.9 (95% CI 1.5 to 5.5) to have pneumonia compared to the highest tertile. Adjusted model for other individual factors (BMI, smoking, education, income) shows a little reduction in HR (2.6 95% CI 1.4 to 5.0); adjusting also for sun exposure at baseline, physical activity, occupation and multivitamin use there was further reduction of HR (2.4 95% CI 1.2 to 4.9). The middle tertile reported HR non statistically significant.
**Conclusion**	There was a dose-dependent increased risk of pneumonia incidence hospitalization in ageing population with low concentrations of vitamin D in blood; the high lost at follow-up could be a bias.

Barrett-Connor 2012

**Biomarker**	**25(OH)D**
**Outcomes**	**Bone Mineral density (BMD)**<br>**Bone loss**<br>**Fracture risk**
**Categories of vitamin D levels**	Cut-off 20 ng/mL<br>Reference: lowest quartiles (<20 ng/mL)
**Results**	Men in the lowest quartile of vitamin D did not have different BMD and bone loss respect to men with higher vitamin D levels at baseline and normal levels of bioavailable estradiol (BioE) and sex hormone binding globulin (SHBG). The greatest hip bone loss (%) was observed in men with low BioE and low vitamin D levels at baseline examination. Similarly, there were not differences in fractures risk in relation to vitamin D levels at baseline. HRs for nonspine fracture and major osteoporotic fracture were not statistically significant for the lowest vitamin D levels concentration, also in adjusted models.
**Conclusion**	Vitamin D deficiency with normality in SHBG was not associated to BMD, bone loss and fractures risks. This study reported that vitamin deficiency with low estrogen and/or high SHBG could improve prediction of osteoporotic markers.<br>Limitations of this study were race homogeneity of cohort that may not be generalizable results to other population. Results were inconsistent to prove predictivity ability of vitamin D for bone health.

Brøndum-Jacobsen 2012

**Biomarker**	**25(OH)D**
**Outcomes**	**Ischemic Heart Disease (IHD)**<br>**Myocardial Infarction (MI)**<br>**Early Death**<br>**Nonfatal ischemic heart diseases**<br>**Nonfatal myocardial infarction**<br>**Fatal ischemic heart disease/myocardial infarction**
**Categories of vitamin D levels**	Quartiles (14-23 nmol/L; 28-46 nmol/L; the upper two 47-71 nmol/L), and the first quartile divided in three categories (7.5–12 nmol/L; 13–17 nmol/L; 18–26 nmol/L); clinical cut points (severe vitamin D deficiency as <25.0 nmol/L, moderate vitamin D deficiency as 25.0–49.9 nmol/L; vitamin D insufficiency as 50.0–74.9 nmol/L, and optimal vitamin D as ≥75.0 nmol/L)<br>Reference: 50.8-112.8 nmol/L in the percentile categories; ≥75 nmol/L in the clincal categories
**Results**	**Percentile categories.** In age-and sex adjusted analysis, subjects in the lowest percentile of vitamin D concentrations (7.5-12 nmol/L) had a major risk (70%) of IHDs, MI (99%), early death (88%), and fatal events for IHDs/MI (122%) in comparison to the two upper quartiles. Risk decreased with the increase of vitamin D levels until 20%, 18%, 19%, and 28% more in subjects with 28-46 nmol/L for IHD, MI, early death, and fatal events (for IHD/MI) respectively. Non significative associations were found for non-fatal events.<br>In multivariable adjusted analysis (for sex, physical activity, smoking, and diabetes mellitus, age, body mass index, pack-years smoked, alcohol consumption, plasma total cholesterol, high-density lipoprotein cholesterol, systolic blood pressure, and estimated glomerular filtration rate), article reports similar trends but with minor risk already for the lowest level concentrations of vitamin D; associations with MI, and fatal events lost significance, except for subjects in the two lower percentile. There were no significative associations with non-fatal events.<br>**Clinical categories of vitamin D cut-points**. In age-and sex adjusted analysis, the lowest category (<25 nmol/L) reported major statistically significant risks of 52%, 72%, 42%, and 110% in comparison to the upper category (≥75 nmol/L) for IHDs, MI, early death, and fatal events (IHMs/MI), respectively. The second category (25-49.9 nmol/L) reported significant risks of 25%, 31%, and 55% for IHDs, MI, and fatal events (IHDs/MI) respectively. All other associations were not statistically significant. While the third category (50-74.99 nmol/L) reported a protective association with early death in comparison to the highest one (HR 0.91 95% CI 0.83 to 1.00), there was a negative trends for fatal events (all vitamin D categories were associated to the outcome change from 110% to 30% of risk more than the highest category with the incresed of vitamin D level concentrations). All analysis for nonfatal events for IHDs and MI were non-statistically significant with HRs close by 1.<br>In multivariable adjusted analysis only the lowest category of vitamin D level concentrations were statistically associated to outcomes (29% for IHDs, 49% for MI, 37% for early death, and 53% for fatal events) in comparison to the highest, while significance there was not for nonfatal events.

**Conclusion**	The inverse association of vitamin D level concentrations at baseline with fatal events for IHDs and MI, and not with nonfatal events could indicate that low vitamin D concentration is indicator of a poor general health. The inverse association between vitamin D and IHDs, MI, and early death is robust.

Cauley 2010

**Biomarker**	**25(OH)D**
**Outcomes**	**Hip fractures**<br>**Non-spine Fractures**
**Categories of vitamin D levels**	Quartiles 3.13 – 19 ng/ml; 19.0 – 27.9 ng/ml; 25.1- 27.9 ng/ml; 27.9 ng/ml<br>Cut-off deficiency < 20 ng/mL; insufficiency 21-30 ng/mL, sufficiency > 30 ng /mL<br>25(OH)D levels used as continuous variable (SD)<br>Reference: Highest quartile
**Results**	The mean 25(OH)D was 24.55 ng/mL in non-spine fracture subjects, 21.15 ng/mL in hip fracture subjects, and 25.15 ng/mL in controls. One SD decrease in total 25(OH)D was associated with an increased risk of hip fracture (multivariate HR ¼ 1.60; 95% CI 1.18–2.17). Compared with men in the top quartile of total 25(OH)D (>28 ng/ml), the HR of hip fracture was 2.36 (95% CI 1.08–5.15) for men in the lowest quartile. There was no evidence of association between vitamin D and non-spine fracture in a model stratified by age.
**Conclusion**	The results of the study suggest that lower vitamin D concentrations are associated with higher risk of hip fracture in older men. No evidence for non-spine fracture.

Heath 2017

**Biomarker**	**25(OH)D**
**Outcomes**	**All-cause mortality**
**Categories of vitamin D levels**	Quintiles (median 30.1 nmol/L, 41 nmol/L, 51.4 nmol/L, 60.7 nmol/L, 76.2 nmol/L)<br>Reference: lowest quintile (median 30.1 nmol/L)
**Results**	HRs for increasing levels of vitamin D concentration in comparison to the lowest quintile were statistically significant over the forth quintile (≥60.7 nmol/L). The highest quintiles (IV and V) resulted protective for all-causes mortality (HRs 0.77 CI 95% 0.62 to 0.97 and 0.68 CI 95% 0.54 to 0.85, respectively for IV and V quintile vs. the I quintile). Estimates were unchanged after exclusion of outliers, and splines trend did not fit better than linear one.
**Conclusion**	Higher levels of vitamin D in adult age were moderately associated to lesser risk of death during a mean of 13.7 (±2.2) years of follow-up. Authors controlled for confounding (history of disease and risk factors) and cannot excluded possibility of residual confounding factors. Exclusion from analysis people with levels concentration of vitamin D above 100 nmol/L, it was unlikey find a U-shape or J-shaped curve between vitamin D status and risk of mortality.

Hirani 2018

**Biomarker**	**25(OH)D**<br>**1.25(OH)D**
**Outcomes**	**Sarcopenia**
**Categories of vitamin D levels**	Quartiles 25(OH)D (<40 nmol/L, 40–52.9 nmol/L, 53–68.9 nmol/L, ≥69nmol/L), 1,25(OH)D (<62 pmol/L, 62–96.9 pmol/L, 97–145.9 pmol/L, ≥146pmol/L)<br>Reference: 25(OH)D (≥69nmol/L), 1.25(OH)D (≥146pmol/L)
**Results**	Lowest quartiles of 25(OH)D (<40 nmol/L) and 1.25(OH)D (< 62 pmol/L) were associated to increase risk of sarcopenia in comparison to each referent categories in unadjusted and adjusted models; the multivariate adjusted models (for age, season, socio-economic factors, life style, healthy status, vitamin D supplement use, white cell count, albumin, PTH eGFR, 25/1.25(OH)D) reported ORs of 2.40 (CI 95% 1.02 to 5.64) and 2.23 (CI 95% 1.04 to 4.80) for 25(OH)D and 1.25(OH)D, respectively.
**Conclusion**	Data reported evidence of a relationship between vitamin D levels and incidence of sarcopenia at 2- and 5-year follow-up in men aged 70 years and over. No controlled analysis by outdoor exposition time, weakly of renal function markers used. Analysis was adjusted for missing data.

Holmberg 2017

**Biomarker**	**25(OH)D**
**Outcomes**	**Ischemic Heart Disease (IHD)**
**Categories of vitamin D levels**	Quartiles (based on serum levels of 25-OH-D among controls at baseline: ≤ 22.3 ng/mL, 22.3 - 28.4 ng/mL, 28.4-34.5 ng/mL, ≥34.5 ng/mL; based on month-adjusted 25-OH-D among controls at baseline: ≤-3.72 ng/mL, -3.72 - 1.02 ng/mL, 1.02 - 7.62 ng/mL, ≥7.62 ng/mL)<br>Reference: quartiles (≤ 22.3 ng/mL); month-adjusted (≤-3.72 ng/mL)
**Results**	Adjusted model (systolic blood pressure, s-cholesterol, body mass index and occupation) showed the highest quartile of vitamin D was protective for IHD in comparison to the lowest level in adjusted measured vitamin D, and not in unadjusted one. This was true also in crude ORs. ORs for highest vs lowest quartile were 0.54 (CI 95% 0.32 to 0.90) and 0.46 (CI 95% 0.25 to 0.84) for crude and adjusted model, respectively.
**Conclusion**	Men in highest quartile of vitamin D concentration at baseline had half the risk of IHD respect to men in lowest quartile, after controlling by risk factors. Match between cases and controls were performed only on age, thus the rational for paired tests was limited. No data on dietary or supplements intake were taken into account as well as on physical activity and sun exposure.

Jassal 2010

**Biomarker**	**25(OH)D**<br>**1.25(OH)D**
**Outcomes**	**Cardiovascular mortality (CVm)**
**Categories of vitamin D levels**	25(OH)D levels used as continuous variable (SD in serum 25-OH-D and in log1.25-OH-D)
**Results**	HRs (CI 95%) for CVm per SD in 25(OH)D were non-statistically significant in all unadjusted and adjusted models. There was significance in unadjusted HR for CVm per SD increase in log1.25(OH)D (0.76 CI 95% 0.63 to 0.90).
**Conclusion**	Limitations were related characteristics of selected people observed (Caucasian, upper-middle class, living in temperate climate) that limit generalization to other population (with vitamin D deficiency, for example). Another limitation was probably the technique used for measure exposure, and the fact that lost at follow-up visit could be attenuating any true association.

Khaw 2014

**Biomarker**	**25(OH)D**
**Outcomes**	**All-cause mortality**<br>**Cardiovascular death (CVD)**<br>**Cancer death (CD)**<br>**Respiratory death (RD)**<br>**Cancer**<br>**Cardiovascular events (CVEs)**<br>**Respiratory disease events (RDEs)**<br>**All fracture events (AFEs)**<br>**Hip-fracture events (HFEs)**
**Categories of vitamin D levels**	Clinical cut-off (<30 nmol/L, 30-49 nmol/L, 50-69 nmol/L, 70-89 nmol/L, ≥90 nmol/L)<br>25(OH)D levels used as continuous variable (per 20nmol/L increase)
**Results**	According to a model adjusted for age, sex and month, a 20-nmol/L increase in 25(OH)D was a significant protective factor for total mortality (H.R = 0.89 C.I. 0.86 -0.93 p <0.0001), for cardiovascular disease deaths (HR= 0.88 C.I. 0.83-0.94; p < 0.0001), for respiratory disease deaths (H.R =0.70 0.62- 0.81 p<0.0001), for all fracture events (HR = 0.88 C.I. 0.81 - 0.96 p =0.002), for cardiovascular disease events (H.R. = 0.92 C.I. 0.90-0.95 p< 0.0001), for respiratory disease events (HR = 0.85 C.I. 0.82-0.90 p<0.0001), for cancer death (HR = 0.93 CI 0.88 -0.99), for hip fracture events (HR 0.81 C.I. 0.79-0.94 p = 0.004), and not for incident total cancer (HR= 1.02 C.I. 0.99 -1.06 p = 0.21.<br>According to a multivariate model adjusted for body mass index, smoking, social class, education, physical activity, alcohol in-take, plasma vitamin C, history of cardiovascular disease, diabetes, or cancer, a 20-nmol/L increase in 25(OH)D was a significant protective factor for total mortality (H.R = 0.92 C.I. 0.88 -0.96 p =<0.001), for cardiovascular disease (HR= 0.96 C.I. 0.93 -0.99; p = 0.014), for respiratory disease (H.R =0.89 C.I. 0.85- 0.93), for cancer death (HR = 0. 95 CI 0.89- 1.00), for all fractures events (HR = 0.89 C.I. 0.81 - 0.98), not for incident total cancer (HR= 1.02 CI 0.99 -1.06 p = 0.21),
**Conclusion**	The findings of this study suggest that there is an inverse significant association between higher levels of vitamin D and lower total mortality and incident cardiovascular disease, respiratory disease, and fractures but not total incident cancers. For mortality outcomes, lowest risks were in participants with higher concentrations. This support the hypothesis that moderate increase in population mean concentrations may have potential health benefit.

Licher 2017

**Biomarker**	**25(OH)D**
**Outcomes**	**Dementia (Mini Mental State Examination, Geriatric Mental Schedule organic level)**<br>**Alzheimer's disease (AD)**
**Categories of vitamin D levels**	Tertiles (0-38 nmol/L; 38-64 nmol/L;64-175 nmol/L)<br>Cut-off: (Endocrine Society): deficiency < 50 nmol/L; insufficiency 50-75 nmol/L , sufficiency > 75 nmol /L<br>Cut-off: (NAM): deficiency < 25 nmol/L; insufficiency 25-50 nmol/L , sufficiency > 50 nmol /L<br>Reference: Third Tertile
**Results**	In non-demented patients, lower vitamin D concentrations were associated with higher risk of dementia (adjusted HR 1.10 CI 95% 1.02- 1.20) and AD (adjusted HR. 1.13, CI 95% 1.03- 1.24).
**Conclusion**	Although residual confounders cannot be excluded, the results of the study suggest that lower vitamin D concentrations are associated with augmented risk of dementia, including AD

Lu 2012

**Biomarker**	**25(OH)D**
**Outcomes**	**Mortality for hearth fealure (HF)**<br>**Mortality for cardiovascular disease (CVD)**<br>**Premature all-cause death**
**Categories of vitamin D levels**	Tertile: 20 ng/ml; 20 to 29 ng/ml; 30 ng/ml<br>Reference: Third tertile
**Results**	Multivariate-adjusted Cox model indicated that subjects with serum 25(OH)D levels <20 ng/ml had 2.06 times higher risk (95% confidence interval 1.01 to 4.25) of HF death than those with serum 25(OH)D levels >30 ng/ml (p <0.001) and that subjects with serum 25(OH)D levels <20 ng/ml had 1.61 times higher risk (95% confidence interval 1.37 to 1.88) of HF death than those with serum 25(OH)D levels >30 ng/ml (p <0.001). Hazard ratios for CVD were significantly >1 for people in the lowest tertile (<20 ng/mL) in comparison to the highest (1.52 95% CI 1.29 – 1.79 in adjusted model for age, sex, health status and lifestyle factors). In addition, hazard ratios (95% C.I.) for premature death from all causes were 1.40 (1.17 to 1.68) in subjects with serum 25(OH)D levels <20 ng/ml and 1.11 (0.93 to 1.33) in those with serum 25(OH)D levels of 20 to 29 ng/ml compared to those with serum 25(OH)D levels >30 ng/ml (p <0.001, test for trend).
**Conclusion**	The findings of this study suggest that there was a significant association between lower level of vitamin D and premature all cause death, in particular for HF, but not for middle level of vitamin D.

Looker 2013

**Biomarker**	**25(OH)D**
**Outcomes**	**Major Osteoporotic Fracture (hip, spine, radius, and humerus)**
**Categories of vitamin D levels**	Serum 25OHD was divided into categories that reflected recently recommended thresholds (eg, 30 nmol/L, 50 nmol/L, 75 nmol/L).<br>25(OH)D levels used as continuous variable (SD)
**Results**	Serum 25OHD was a significant predictor of major osteoporotic fracture (in particular hip fracture) in the total sample and among those with less than 10 years of follow-up, but it was not related to risk of either fracture type among those with 10 years of follow-up. Major osteoporotic fracture risk was increased by 26% to 27% for each SD decrease in serum 25OHD among those with less than 10 years of follow-up. Serum 25OHD was significantly related to risk of major osteoporotic fractures as a group and to hip fracture alone in this cohort of older U.S. adults from NHANES III and NHANES 2000–2004. However, this association diminished after 10 years of follow up.
**Conclusion**	The findings of this study suggest that there is a significant association between lower levels of vitamin D and major osteoporotic fractures (in particular hip fracture). This association diminished after 10 years of follow up.

Marques-Vidal 2015

**Biomarker**	**Total 25(OH)D3**<br>**25(OH)D3**
**Outcomes**	**Insulin resistance (IR)**
**Categories of vitamin D levels**	Quartile: Total 25(OH)D3, (3.9–34.0) (34.0–49.7) (49.7–68.2) nmol/L<br>25(OH)D3, (4.3–31.6) (31.6–46.9) (46.9–64.2) (64.2–149) nmol/L<br>Refence: First quartile
**Results**	Participants who developed IR had lower baseline serum concentrations of 25-hydroxyvitamin D3.<br>Multivariable analysis adjusting for month of sampling, age, and sex showed that participants in the first quartile had an augmented risk of developing IR, compared with other quartiles.<br>Similar associations were found between total 25(OH)D3 and incident IR. Further adjustment for body mass index, sedentary status, and smoking didn't show an association between 25(OH)D3, total 25(OH)D3 and the risk of developing IR.
**Conclusion**	The findings of this study suggest that there isn't a significant association between lower levels of vitamin D 's metabolites and the risk of IR. This study has some limitations. Firstly, only 57% of participants were included, which may have reduced statistical power and biased the results. The second limitation is represented by the homogeneity of the sample (the participants were from the same city and sunlight exposure).

Mursu 2015

**Biomarker**	**25(OH)D**
**Outcomes**	**Risk of disease death**
**Categories of vitamin D levels**	Tertile: 32.1<; 32.1–49.4; >49.4 nmol/L<br>Refence: Third tertile
**Results**	The multi-variate-adjusted hazard ratio (HR) for death comparing participants in the lowest to the highest serum 25(OH)D3 tertile was 1.31 (95 % CI 1.07–1.60, p = 0.01). Stratified by the magnesium intake, the higher risk was observed only in the lower magnesium intake median: HR = 1.60 (95 % CI 1.19–2.13, p= 0.002) in the lowest versus the highest 25(OH)D3 tertile.
**Conclusion**	The findings of this study suggest that in this cohort of middle- aged and older men low serum 25(OH)D3 concentration was associated with increased risk of death mainly in those with lower magnesium intake. The most important limit of this study is that the intake of magnesium and serum 25(OH)D3 concentration were measured only at baseline, which potentially may cause misclassification and therefore attenuate the studied associations.

Olsson 2017

**Biomarker**	**25(OH)D**
**Outcomes**	**Dementia**<br>**Cognitive impairment**
**Categories of vitamin D levels**	Tertiles < 50 ; 50- 75 ; > 75 nmol /L<br>Reference : Third tertile
**Results**	Plasma 25(OH)D was not associated with any cognitive outcomes (crude and adjusted HRs and ORs were =1.0 for all continuous exposures). The adjusted (for age, season of blood collection, BMI, education, physical activity, smoking, diabetes, hypertension, hypercholesterolemia, use of vitamin d supplements, and alcohol intake) HR for all-cause dementia was 0.86 (95% CI: 0.58, 1.30) in men in the first tertile compared with them in the third tertile.
**Conclusion**	The findings of this study suggest that levels of vitamin D may not be associated with altered cognitive functioning in the elderly. The limitations of the study include the lack of repeated measurements of plasma 25(OH)D, the lack of information on sun exposure as a covariate, the inclusion of men only, and the small number of men with low concentrations of 25(OH)D.

Platz 2004

**Biomarker**	**25(OH)D**<br>**1.25(OH)D**
**Outcomes**	**Prostate cancer**
**Categories of vitamin D levels**	Quartiles: -<br>Reference: First quartile
**Results**	Mean concentrations of 1.25(OH)D and 25(OH)D were slightly, but not statistically significantly (p = 0.06 and 0.20, respectively), higher in cases (34.3 ± 7.1 pg/ml and 24.6 ± 7.7 ng/ml, respectively) than in controls (33.5 ± 7.1 pg/ml and 23.9 ± 8.2 ng/ml, respectively). The OR of prostate cancer comparing men in the fourth to first quartile of 1.25(OH)D was 1.25 (95% CI: 0.82–1.90, p = 0.16). For 25(OH)D, the OR of prostate cancer comparing the highest and the lowest quartiles was 1.19 (95% CI: 0.79–1.79, p= 0.59). These findings did not vary adjusting by level of the other metabolite, age at diagnosis, family history of prostate cancer, or factors that don't influence 25(OH)D levels.
**Conclusion**	The findings of this study suggest that there is no significant association between lower levels of vitamin D and prostate cancer.

Shimizu 2015

**Biomarker**	**25(OH)D**
**Outcomes**	**Risk of falls**
**Categories of vitamin D levels**	Tertiles <20 ng /mL; 20- 25 ng /mL; > 25 ng /mL<br>Reference : Third tertile
**Results**	Plasma 25(OH)D was associated with risk of falls in Japanese community- dwelling elderly women. Compared with women in the top quartile of total 25(OH)D, the OR was 1.40 for any falls (95% CI 1.01–1.94) and 1.47 (95 % CI 0.93-2.32) for recurrent falls among women in the lowest quartile.
**Conclusion**	The findings of this study suggest that lower levels of vitamin D may be associated with an increased risk of falls in the elderly women. The most important limitations of the study include the lack of repeated measurements of vitamin D and dietary vitamin D intakes, the lack of information on sun exposure, the inclusion of only men, the small size of the population studied.

Swanson 2015

**Biomarker**	**25(OH)D**<br>**1.25(OH)D**
**Outcomes**	**Bone loss at the hip**<br>**Bone loss at the lumbar spine**<br>**Incident non-vertebral fracture**<br>**Incident hip fractures**
**Categories of vitamin D levels**	Quartiles:<br>25(OH)D 3.13-20.90 ng/mL; 20.91-25.90 ng/mL; 26.00- 31-00 ng/mL; 31.10-55.80 ng/Ml<br>1.25(OH)D 8.70- 51.60 pg/mL; 51.70-62.00 pg/mL ; 62.10- 75-10 pg/mL; 75.20 – 142.00 pg/mL<br>SD increase in 25(OH)D and 1.25(OH)D<br>Reference:<br>25(OH)D (3.13-20.90 ng/mL)<br>1.25(OH)D (8.70 – 51.60 pg/mL)
**Results**	In all models, there was a negative association between 25(OH)D levels and bone loss at the hip (-0.66% mean annualized percent change in multivariate model). Association was negative also for bone loss at the lumbar spine, but only for SD increase (trend was not significant for quartiles of 25(OH)D). Associations of 1.25(OH)D with bone loss were non statistically significant.<br>There were linear associations between the two measures of vitamin D and fracture outcomes. Risk of hip fracture was roughly 30% lower for SD increase in 25(OH)D. Analyses by quartiles showed significant associations between the lowest 25(OH)D levels and hip fractures in comparison to the highest category. Both vitamin D metabolites were not associated to non-vertebral fractures. 1.25(OH)D was associated to hip fractures only in Bone Mineral Density-adjusted model, and this was not affected by adjustment for falls or walk speed. The association of 1.25 (OH)D with skeletal outcomes were weaker than those with 25(OH)D.
**Conclusion**	The results of this study don't support the hypothesis that measures of 1.25(OH)D improve the ability to predict adverse skeletal outcomes (such as BMD loss, incident falls, incident non-vertebral fracture) better than 25OHD.

Umehara 2017

**Biomarker**	**1.25(OH)D**
**Outcomes**	**All cause death**<br>**Cardiovascular death**<br>**Respiratory death**<br>**Cancer death**
**Categories of vitamin D levels**	Quartile: <54; 54-65.3; 65.4 – 78.1: >/= 78.2 pg/ml<br>Reference: Fourth quartile
**Results**	The multivariable-adjusted and the sex-age adjusted hazard ratio (HR) for all-cause death increased significantly with lower serum 1.25(OH)D levels (HR 1.54 95% CI 1.18–2.01). This study found a similar association for cardiovascular and respiratory infection death (both P for trend <0.01), but not for cancer death or death from other causes. In the stratified analysis, the association between lower serum 1.25(OH)D levels and the risk of respiratory infection death was stronger in subjects with kidney dysfunction with an estimated glomerular filtration rate (eGFR) <60mL/min/1.73m2.
**Conclusion**	The findings of this study suggest that there is a significant association between lower levels of vitamin D and all cause death, in particular cardiovascular and respiratory infection death. Among participants with kidney dysfunction, lower levels of vitamin D are greatly associated with augmented risk of respiratory infection death. Limits were the single measurement of 1,25(OH)2D levels at baseline, and the reverse causality (the serum vitamin D in subjects with chronic diseases would be influenced by drug and lifestyle).

Vàzquez-Oliva 2018

**Biomarkers**	**25(OH)D**
**Outcomes**	**Cardiovascular events (angina, fatal or nonfatal myocardial infarction, coronary death)**
**Categories of vitamin D levels**	25(OH)D levels used as continuous variable (SD)
**Results**	In multivariate analyses adjusted for systolic and diastolic blood pressure, total cholesterol, HDL cholesterol, diabetes, smoking status, vitamin D increase (SD) is not associated with coronary events (p = 0.334). In a statistical model adjusted for age and sex, vitamin D increase (SD) has a cardio protective effect (HR 0.71 95 % CI 0.58- 0.88)
**Conclusion**	Vitamin D is not associated with coronary events; the seasonal variation may have influenced the results obtained.
