# Supplementary material for: Vitamin D as a Biomarker of Ill-Health among the Over-50s: A Systematic Review of Cohort Studies
Source: Nutrients. 2019 Oct 6;11(10):2384. doi: 10.3390/nu11102384 (PMC6836048; doi:10.3390/nu11102384)
Supplement: Supplementary file 1 [file nutrients-11-02384-s001.zip › Table S1 Search strategy.pdf]

Table S1: Search strategy

1) **Search 1**
   Database PubMed
   Date 6th February 2019
   Total records 2194
   String:

   (vitamin d[MeSH Terms] OR vitamin d deficiency[MeSH Terms] OR 25-hydroxyvitamin d[MeSH Terms] OR “vitamin d”[Title/Abstract] OR “25-hydroxyvitamin d”[Title/Abstract] OR “25(OH)D” [Title/Abstract] OR “1,25(OH)2D”[Title/Abstract] OR “1,25 dihydroxyvitamin d”[Title/Abstract] OR 1,25 dihydroxyvitamin d[MeSH Terms]) AND (aged[MeSH Terms] OR aging[MeSH Terms] OR "Aged, 80 and over"[Mesh] OR "old age people" OR elder* OR adult* OR retired OR ancient*) AND (healthy aging[MeSH Terms] OR Quality-Adjusted Life Years[MeSH Terms] OR longevity[MeSH Terms] OR “healthy ag*” OR “healthy status” OR “health status” OR “ill health” OR longevity OR mortality OR “all-causes death*” OR death* OR diseas* OR “physical function*” OR “cognitive function*” OR “cognitive impairment” OR “social function*” OR wellbeing OR well-being OR “quality of life” OR “aging status” OR “ageing status” OR balance OR “physical performanc*” OR frailt*) NOT supplement* NOT therapy[MeSH Terms] NOT patients[MeSH Terms] NOT patient*[Title/Abstract] NOT ill* NOT "medicin* use" NOT “drug* use” NOT “medicin* therap*” NOT “drug* therap*” NOT treat* NOT care NOT cure Filters: Publication date from 2000/01/01 to 2019/12/31

**#25**	**#24 Filters: Publication date from 2000/01/01 to 2019/12/31, 2194**
**#24**	(#17 AND #1 AND #7) NOT supplement* NOT therapy[MeSH Terms] NOT patients[MeSH Terms] NOT patient*[Title/Abstract] NOT ill* NOT "medicin* use" NOT “drug* use”NOT “medicin* therap*”NOT “drug* therap*”NOT treat* NOT care NOT cure, 3294
**#23**	(#17 AND #1 AND #7) NOT supplement*, 14959
**#22**	Search NOT therapy[MeSH Terms] NOT patients[MeSH Terms] NOT patient*[Title/Abstract] NOT ill* NOT "medicin* use" NOT “drug* use” NOT “medicin* therap*”NOT “drug* therap*”NOT treat* NOT care NOT cure, 1339825
**#21**	(#17 AND #1 AND #7) NOT (#20 OR #2), 837
**#20**	Search supplement*, 310371
**#19**	#17 AND #1 AND #7, 19991
**#18**	#17 AND #1, 30405
**#17**	Search (vitamin d[MeSH Terms] OR vitamin d deficiency[MeSH Terms] OR 25-hydroxyvitamin d[MeSH Terms] OR “vitamin d”[Title/Abstract] OR “25-hydroxyvitamin d”[Title/Abstract] OR “25(OH)D”[Title/Abstract] OR “1,25(OH)2D”[Title/Abstract] OR “1,25 dihydroxyvitamin d “[Title/Abstract] OR 1,25 dihydroxyvitamin d[MeSH Terms]), 87537
**#16**	Search (vitamin d[MeSH Terms] OR vitamin d deficiency[MeSH Terms] OR 25-hydroxyvitamin d[MeSH Terms] OR “vitamin d”[Title/Abstract] OR “25-hydroxyvitamin d”[Title/Abstract] OR “25(OH)D”[Title/Abstract] OR “1,25(OH)2D”[Title/Abstract] OR “1,25 dihydroxyvitamin d “[Title/Abstract] OR 1,25 dihydroxyvitamin d[MeSH Terms]) Filters: Observational Study, 538
**#15**	#14 Filters: Publication date from 2000/01/01 to 2019/12/31,4532
**#14**	#13 Filters: Observational Study, 4532

**#13**	Search ((aged[MeSH Terms] OR aging[MeSH Terms] OR “Aged, 80 and over”[Mesh] OR “old age people”OR elder* OR adult* OR retired OR ancient*) NOT therapy[MeSH Terms] NOT patients[MeSH Terms] NOT “medicin* use”NOT “drug* use”NOT “medicin* therap*”NOT “drug* therap*”NOT treat* NOT care NOT cure AND (untreat*) AND (vitamin d[MeSH Terms] OR vitamin d deficiency[MeSH Terms] OR 25-hydroxyvitamin d[MeSH Terms] OR “vitamin d”OR “25-hydroxyvitamin d”OR “25(OH)D”) AND (association[MeSH Terms] OR associat* OR predict*) AND (“healthy aging”[MeSH Terms] OR “Quality-Adjusted Life Years”[MeSH Terms] OR longevity[MeSH Terms] OR “healthy ag*”OR “healthy status”OR “health status”OR “ill health”OR longevity OR mortality OR “all-causes death*”OR death* OR diseas* OR “physical function*”OR “cognitive function*”OR “cognitive impairment”OR “social function*”OR wellbeing OR well-being) OR “quality of life”OR “aging status”OR “ageing status”OR balance OR “physical performanc*”OR frailt*)), 640487
**#9**	Search ((aged[MeSH Terms] OR aging[MeSH Terms] OR "Aged, 80 and over"[Mesh] OR "old age people" OR elder* OR adult* OR retired OR ancient*) NOT therapy[MeSH Terms] NOT patients[MeSH Terms] NOT "medicin* use" NOT “drug* use”NOT “medicin* therap*”NOT “drug* therap*”NOT treat* NOT care NOT cure AND untreat* AND (vitamin d[MeSH Terms] OR vitamin d deficiency[MeSH Terms] OR 25-hydroxyvitamin d[MeSH Terms] OR vitamin d 3[MeSH Terms] OR “vitamin d”OR “vitamin d3”OR “25-hydroxyvitamin d”OR “25(OH)D”OR 1,25 dihydroxyvitamin d3[MeSH Terms] OR "1,25 gihydroxyvitamin d*" OR "1,25(OH)2D3") AND (association[MeSH Terms] OR associat* OR predict*) AND (healthy aging[MeSH Terms] OR Quality-Adjusted Life Years[MeSH Terms] OR longevity[MeSH Terms] OR “healthy ag*”OR “healthy status”OR “health status”OR “ill health”OR longevity OR mortality OR “all-causes death*”OR death* OR diseas* OR “physical function*”OR “cognitive function*”OR “cognitive impairment”OR “social function*”OR wellbeing OR well-being OR “quality of life”OR “aging status”OR “ageing status”OR balance OR “physical performanc*”OR frailt*)))), 27
**#8**	(#1 AND #5 AND #6 AND #7) NOT #2, 15
**#7**	Search (“healthy aging”[MeSH Terms] OR “Quality-Adjusted Life Years”[MeSH Terms] OR longevity[MeSH Terms] OR “healthy ag*”OR “healthy status”OR “health status”OR “ill health”OR longevity OR mortality OR “all-causes death*”OR death* OR diseas* OR “physical function*”OR “cognitive function*”OR “cognitive impairment”OR “social function*”OR wellbeing OR well-being OR “quality of life”OR “aging status”OR “ageing status”OR balance OR “physical performanc*”OR frailt*), 11981403
**#6**	Search (association[MeSH Terms] OR associat* OR predict*), 5191641
**#5**	Search (vitamin d[MeSH Terms] OR vitamin d deficiency[MeSH Terms] OR 25-hydroxyvitamin d 2[MeSH Terms] OR 25-hydroxyvitamin d[MeSH Terms] OR vitamin d 3[MeSH Terms] OR “vitamin d”OR “vitamin d3”OR “25-hydroxyvitamin d”OR “25(OH)D”OR 1,25 dihydroxyvitamin d3[MeSH Terms] OR "1.25 gihydroxyvitamin d*" OR "1.25(OH)2D3"), 94347
**#4**	Search 1,25 dihydroxyvitamin d3[MeSH Terms], 13805
**#3**	Search untreat*, 164004
**#2**	Search (NOT therapy[MeSH Terms] NOT patients[MeSH Terms] NOT “vitamin d2”NOT "medicin* use" NOT “drug* use”NOT “medicin* therap*”NOT “drug* therap*”NOT treat* NOT care NOT cure), 1700995
**#1**	Search (aged[MeSH Terms] OR aging[MeSH Terms] OR "Aged, 80 and over"[Mesh] OR "old age people" OR elder* OR adult* OR retired OR ancient*), 6851141

2) **Search 2 (longevity and mortality)**
Database PubMed
Date 28th March 2019
Total records 219
String:

(((((vitamin d[MeSH Terms] OR vitamin d deficiency[MeSH Terms] OR 25-hydroxyvitamin d[MeSH Terms] OR “vitamin d”[Title/Abstract] OR “25-hydroxyvitamin d”[Title/Abstract] OR “25(OH)D”[Title/Abstract] OR “1,25(OH)2D”[Title/Abstract] OR “1,25 dihydroxyvitamin d”[Title/Abstract] OR

1,25 dihydroxyvitamin d[MeSH Terms])) AND ((aged[MeSH Terms] OR aging[MeSH Terms] OR "Aged, 80 and over"[Mesh] OR "old age people" OR elder* OR adult* OR retired OR ancient*))) AND ((longevity[MeSH Terms] " OR longevity OR mortality OR "all-causes death*" OR death* OR surviv*))) NOT ((supplement* OR therapy[MeSH Terms] OR patients[MeSH Terms] OR patient*[Title/Abstract] OR therap*))) NOT ((cross-sectional[Title/Abstract] OR trial*[Title/Abstract])) Filters: Humans

**#13**	**#1 AND #2 AND #3 NOT #10 NOT #11 Filters: Humans, 219**
**#12**	#1 AND #2 AND #3 NOT #10 NOT #11, 261
**#11**	Search (OR cross-sectional[Title/Abstract] OR trial*[Title/Abstract]), 1231368
**#10**	Search (OR supplement* OR therapy[MeSH Terms] OR patients[MeSH Terms] OR patient*[Title/Abstract] OR therap*), 10127305
**#9**	Search (NOT cross-sectional[Title/Abstract] NOT trial*[Title/Abstract]), 294801
**#8**	Search (NOT supplement* NOT therapy[MeSH Terms] NOT patients[MeSH Terms] NOT patient*[Title/Abstract] NOT therap*), 180940
**#7**	#1 AND #2 AND #3, 1399
**#6**	#1 AND #2 AND #3 NOT #4 Schema: all, 0
**#5**	#1 AND #2 AND #3 NOT #4, 0
**#4**	Search (NOT supplement* NOT therapy[MeSH Terms] NOT patients[MeSH Terms] NOT patient*[Title/Abstract] NOT ill* NOT "medicin* use" NOT "drug* use" NOT "medicin* therap*"NOT "drug* therap*"NOT treat* NOT care NOT cure), 116372
**#3**	Search (longevity[MeSH Terms] "OR longevity OR mortality OR "all-causes death*"OR death* OR surviv*), 1948735
**#2**	Search (aged[MeSH Terms] OR aging[MeSH Terms] OR "Aged, 80 and over"[Mesh] OR "old age people" OR elder* OR adult* OR retired OR ancient*), 6919264
**#1**	Search vitamin d[MeSH Terms] OR vitamin d deficiency[MeSH Terms] OR 25-hydroxyvitamin d[MeSH Terms] OR "vitamin d"[Title/Abstract] OR "25-hydroxyvitamin d"[Title/Abstract] OR "25(OH)D"[Title/Abstract] OR "1,25(OH)2D"[Title/Abstract] OR "1,25 dihydroxyvitamin d"[Title/Abstract] OR 1,25 dihydroxyvitamin d[MeSH Terms], 88545

3) **Search 3 (cardiovascular, coronary, and cerebrovascular diseases)**
Database PubMed
Date 28th March 2019
Total records 255
String:

((((((vitamin d[MeSH Terms] OR vitamin d deficiency[MeSH Terms] OR 25-hydroxyvitamin d[MeSH Terms] OR "vitamin d"[Title/Abstract] OR "25-hydroxyvitamin d"[Title/Abstract] OR "25(OH)D" [Title/Abstract] OR "1,25(OH)2D"[Title/Abstract] OR "1,25 dihydroxyvitamin d"[Title/Abstract] OR 1,25 dihydroxyvitamin d[MeSH Terms])) AND (((aged[MeSH Terms] OR aging[MeSH Terms] OR "Aged, 80 and over"[Mesh] OR "old age people" OR elder* OR adult* OR retired OR ancient*)) AND ((((("cardiovascular diseas*" OR "cardio heart diseas*" OR "coronary diseas*" OR "ischemic" OR "infarction" OR "atrial fibrillation" OR "angina" OR "cerebrovascular diseas*" OR "stroke")) NOT ((supplement* OR therapy[MeSH Terms] OR patients[MeSH Terms] OR patient*[Title/Abstract] OR therap* OR cross-sectional[Title/Abstract] OR trial*[Title/Abstract] OR pregnan* OR child* OR adolescent* OR gene*)) Filters: Publication date from 2000/01/01 to 2019/03/01; Humans

**#14**	**#13 Filters: Publication date from 2000/01/01 to 2019/03/01, Humans, 255**
**#13**	(#1 AND #2 AND #9 AND Humans[Mesh]) NOT #12, 297
**#12**	Search (supplement* OR therapy[MeSH Terms] OR patients[MeSH Terms] OR patient*[Title/Abstract] OR therap* OR cross-sectional[Title/Abstract] OR trial*[Title/Abstract] OR pregnan* OR child* OR adolescent* OR gene*), 14133250
**#11**	(#1 AND #2 AND #9 AND Humans[Mesh]) NOT #7 Filters: Publication date from 2000/01/01 to 2019/03/01, Humans",283
**#10**	(#1 AND #2 AND #9 AND Humans[Mesh]) NOT #7 Filters: Humans, 325
**#9**	Search ((("cardiovascular diseas*" OR "cardio heart diseas*" OR "coronary diseas*" OR "ischemic" OR "infarction" OR "atrial fibrillation" OR "angina" OR "cerebrovascular diseas*" OR "stroke")) AND Humans[Mesh]) Filters: Humans, 1263250
**#8**	(#1 AND #2 AND #3 AND Humans[Mesh]) NOT #7 Filters: Humans, 310

**#7**	Search (((supplement* OR therapy[MeSH Terms] OR patients[MeSH Terms] OR patient*[Title/Abstract] OR therap* OR cross-sectional[Title/Abstract] OR trial*[Title/Abstract] OR pregnan* OR child* OR adolescent*) Filters: Humans, 10296659
**#6**	(#1 AND #2 AND #3 AND Humans[Mesh]) NOT #5 Filters: Humans, 404
**#5**	Search (supplement* OR therapy[MeSH Terms] OR patients[MeSH Terms] OR patient*[Title/Abstract] OR therap* OR cross-sectional[Title/Abstract] OR trial*[Title/Abstract]) Filters: Humans, 8423982
**#4**	(#1 AND #2 AND #3 AND Humans[Mesh]) Filters: Humans, 2096
**#3**	Search ("cardiovascular diseas*" OR "cardio heart diseas*" OR "ischemic" OR "infarction" OR "atrial fibrillation" OR "angina" OR "cerebrovascular diseas*" OR "stroke") Filters: Humans, 1214268
**#2**	Search (aged[MeSH Terms] OR aging[MeSH Terms] OR "Aged, 80 and over"[Mesh] OR "old age people" OR elder* OR adult* OR retired OR ancient*) Filters: Humans, 6381469
**#1**	Search (vitamin d[MeSH Terms] OR vitamin d deficiency[MeSH Terms] OR 25-hydroxyvitamin d[MeSH Terms] OR “vitamin d”[Title/Abstract] OR “25-hydroxyvitamin d”[Title/Abstract] OR “25(OH)D” [Title/Abstract] OR “1,25(OH)2D”[Title/Abstract] OR “1,25 dihydroxyvitamin d”[Title/Abstract] OR 1,25 dihydroxyvitamin d[MeSH Terms]) Filters: Humans, 65034

4) **Search 4 (diabetes and metabolic diseases)**
Database PubMed
Date 28th March 2019
Total records 218
String:

(((((vitamin d[MeSH Terms] OR vitamin d deficiency[MeSH Terms] OR 25-hydroxyvitamin d[MeSH Terms] OR “vitamin d”[Title/Abstract] OR “25-hydroxyvitamin d”[Title/Abstract] OR “25(OH)D” [Title/Abstract] OR “1,25(OH)2D”[Title/Abstract] OR “1,25 dihydroxyvitamin d”[Title/Abstract] OR 1,25 dihydroxyvitamin d[MeSH Terms]))) AND ((aged[MeSH Terms] OR aging[MeSH Terms] OR "Aged, 80 and over"[Mesh] OR "old age people" OR elder* OR adult* OR retired OR ancient*))) AND ((diabetes OR "diabetes mellitus" OR "type 2 diabetes" OR "T2DM" OR "type 2 DM" OR "metabolic syndrome*"))) NOT ((supplement* OR therapy[MeSH Terms] OR patients[MeSH Terms] OR patient*[Title/Abstract] OR therap* OR cross-sectional[Title/Abstract] OR trial*[Title/Abstract] OR pregnan* OR child* OR adolescent* OR gene* Or mendelian OR prevalenc)) Filters: Publication date from 2000/01/01 to 2019/03/01; Humans

**#15**	**#14 Filters: Humans, 218**
**#14**	#13 Filters: Publication date from 2000/01/01 to 2019/03/01, 255
**#13**	(#1 AND #2 AND #3) NOT #12, 290
**#12**	Search (supplement* OR therapy[MeSH Terms] OR patients[MeSH Terms] OR patient*[Title/Abstract] OR therap* OR cross-sectional[Title/Abstract] OR trial*[Title/Abstract] OR pregnan* OR child* OR adolescent* OR gene* Or mendelian OR prevalenc*), 14283922
**#11**	#10 Filters: Humans, 276
**#10**	#9 Filters: Publication date from 2000/01/01 to 2019/03/01, 321
**#9**	#5 NOT #8, 357
**#8**	Search mendelian, 11692
**#5**	(#1 AND #2 AND #3) NOT #4, 361
**#7**	#6 Filters: Publication date from 2000/01/01 to 2019/03/01, Humans, 279
**#6**	(#1 AND #2 AND #3) NOT #4 Filters: Publication date from 2000/01/01 to 2019/03/01, 324
**#4**	Search (supplement* OR therapy[MeSH Terms] OR patients[MeSH Terms] OR patient*[Title/Abstract] OR therap* OR cross-sectional[Title/Abstract] OR trial*[Title/Abstract] OR pregnan* OR child* OR adolescent* OR gene*), 14133250
**#3**	Search (diabetes OR "diabetes mellitus" OR "type 2 diabetes" OR "T2DM" OR "type 2 DM" OR "metabolic syndrome*"), 691310
**#2**	Search (aged[MeSH Terms] OR aging[MeSH Terms] OR "Aged, 80 and over"[Mesh] OR "old age people" OR elder* OR adult* OR retired OR ancient*), 6919264
**#1**	Search (vitamin d[MeSH Terms] OR vitamin d deficiency[MeSH Terms] OR 25-hydroxyvitamin d[MeSH Terms] OR “vitamin d”[Title/Abstract] OR “25-hydroxyvitamin d”[Title/Abstract] OR “25(OH)D” [Title/Abstract] OR “1,25(OH)2D”[Title/Abstract] OR “1,25 dihydroxyvitamin d”[Title/Abstract] OR 1,25 dihydroxyvitamin d[MeSH Terms]), 88545

5) **Search 5 (cancer)**
Database PubMed
Date 29th March 2019
Total records 167
String:

((((((vitamin d[MeSH Terms] OR vitamin d deficiency[MeSH Terms] OR 25-hydroxyvitamin d[MeSH Terms] OR “vitamin d”[Title/Abstract] OR “25-hydroxyvitamin d”[Title/Abstract] OR “25(OH)D” [Title/Abstract] OR “1,25(OH)2D”[Title/Abstract] OR “1,25 dihydroxyvitamin d”[Title/Abstract] OR 1,25 dihydroxyvitamin d[MeSH Terms]))) AND ((aged[MeSH Terms] OR aging[MeSH Terms] OR "Aged, 80 and over"[Mesh] OR "old age people" OR elder* OR adult* OR retired OR ancient*))) AND ((cancer* OR tumor* OR tumour* OR melanoma OR neoplasm*))) NOT ((supplement* OR therapy[MeSH Terms] OR patients[MeSH Terms] OR patient*[Title/Abstract] OR therap* OR cross-sectional[Title/Abstract] OR trial*[Title/Abstract] OR pregnan* OR child* OR adolescent* OR gene* OR mendelian OR prevalenc* OR protocol[Title/Abstract]))) AND ((cohort OR longitudinal OR prospectiv* OR “nested case-control”)) Filters: Publication date from 2000/01/01 to 2019/03/01; Humans

**#9**	**#7 NOT #8 Filters: Publication date from 2000/01/01 to 2019/03/01, Humans", 167**
**#8**	Search (cohort OR longitudinal OR prospectiv* OR “nested case-control”), 2356311
**#7**	#6 Filters: Humans, 443
**#6**	#5 Filters: Publication date from 2000/01/01 to 2019/03/01, 466
**#5**	(#1 AND #2 AND #3) NOT #4, 680
**#4**	Search (supplement* OR therapy[MeSH Terms] OR patients[MeSH Terms] OR patient*[Title/Abstract] OR therap* OR cross-sectional[Title/Abstract] OR trial*[Title/Abstract] OR pregnan* OR child* OR adolescent* OR gene* OR mendelian OR prevalenc* OR protocol[Title/Abstract]), 14368617
**#3**	Search (cancer* OR tumor* OR tumour* OR melanoma OR neoplasm*), 3916485
**#2**	Search (aged[MeSH Terms] OR aging[MeSH Terms] OR "Aged, 80 and over"[Mesh] OR "old age people" OR elder* OR adult* OR retired OR ancient*), 6919264
**#1**	Search (vitamin d[MeSH Terms] OR vitamin d deficiency[MeSH Terms] OR 25-hydroxyvitamin d[MeSH Terms] OR “vitamin d”[Title/Abstract] OR “25-hydroxyvitamin d”[Title/Abstract] OR “25(OH)D” [Title/Abstract] OR “1,25(OH)2D”[Title/Abstract] OR “1,25 dihydroxyvitamin d”[Title/Abstract] OR 1,25 dihydroxyvitamin d[MeSH Terms]), 88545

6) **Search 6 (respiratory diseases)**
Database PubMed
Date 28th March 2019
Total records 15
String:

((((((((vitamin d[MeSH Terms] OR vitamin d deficiency[MeSH Terms] OR 25-hydroxyvitamin d[MeSH Terms] OR “vitamin d”[Title/Abstract] OR “25-hydroxyvitamin d”[Title/Abstract] OR “25(OH)D” [Title/Abstract] OR “1,25(OH)2D”[Title/Abstract] OR “1,25 dihydroxyvitamin d”[Title/Abstract] OR 1,25 dihydroxyvitamin d[MeSH Terms]))) AND ((aged[MeSH Terms] OR aging[MeSH Terms] OR "Aged, 80 and over"[Mesh] OR "old age people" OR elder* OR adult* OR retired OR ancient*))) AND (("lung function*" OR "pulmonary function*" OR "pulmonary diseas*" OR "lung diseas*")))) AND ((cohort OR longitudinal OR prospectiv*)))) NOT ((supplement* OR therapy[MeSH Terms] OR patients[MeSH Terms] OR patient*[Title/Abstract] OR therap* OR cross-sectional[Title/Abstract] OR trial*[Title/Abstract] OR pregnan* OR child* OR adolescent* OR gene* OR mendelian OR prevalenc* OR protocol[Title/Abstract]))

**#8**	**#7 NOT #5, 15**

**#7**	#6 AND #4, 222
**#6**	#1 AND #2 AND #3, 657
**#5**	Search (supplement* OR therapy[MeSH Terms] OR patients[MeSH Terms] OR patient*[Title/Abstract] OR therap* OR cross-sectional[Title/Abstract] OR trial*[Title/Abstract] OR pregnan* OR child* OR adolescent* OR gene* OR mendelian OR prevalenc* OR protocol[Title/Abstract]), 14368617
**#4**	Search (cohort OR longitudinal OR prospectiv*), 2356311
**#3**	Search ("lung function*" OR "pulmonary function*" OR "pulmonary diseas*" OR "lung diseas*"), 540157
**#2**	Search (aged[MeSH Terms] OR aging[MeSH Terms] OR "Aged, 80 and over"[Mesh] OR "old age people" OR elder* OR adult* OR retired OR ancient*), 6919264
**#1**	Search (vitamin d[MeSH Terms] OR vitamin d deficiency[MeSH Terms] OR 25-hydroxyvitamin d[MeSH Terms] OR “vitamin d”[Title/Abstract] OR “25-hydroxyvitamin d”[Title/Abstract] OR “25(OH)D” [Title/Abstract] OR “1,25(OH)2D”[Title/Abstract] OR “1,25 dihydroxyvitamin d”[Title/Abstract] OR 1,25 dihydroxyvitamin d[MeSH Terms]), 88545

7) **Search 7 (Osteoporosis)**
Database PubMed
Date 28th March 2019
Total records 126
String:

(((((((vitamin d[MeSH Terms] OR vitamin d deficiency[MeSH Terms] OR 25-hydroxyvitamin d[MeSH Terms] OR “vitamin d”[Title/Abstract] OR “25-hydroxyvitamin d”[Title/Abstract] OR “25(OH)D” [Title/Abstract] OR “1,25(OH)2D”[Title/Abstract] OR “1,25 dihydroxyvitamin d”[Title/Abstract] OR 1,25 dihydroxyvitamin d[MeSH Terms]))) AND ((aged[MeSH Terms] OR aging[MeSH Terms] OR "Aged, 80 and over"[Mesh] OR "old age people" OR elder* OR adult* OR retired OR ancient*))) AND ((osteoporosis OR "bone loss" OR "bone mineral density"))) AND ((cohort OR longitudinal OR prospectiv* OR “nested case-control”)))) NOT ((supplement* OR therapy[MeSH Terms] OR patients[MeSH Terms] OR patient*[Title/Abstract] OR therap* OR cross-sectional[Title/Abstract] OR trial*[Title/Abstract] OR pregnan* OR child* OR adolescent* OR infant* OR gene* OR mendelian OR prevalenc* OR protocol[Title/Abstract])) Filters: Publication date from 2000/01/01 to 2019/03/01; Humans

**#10**	**#9 Filters: Publication date from 2000/01/01 to 2019/03/01, Humans, 126**
**#9**	#7 NOT #8, 157
**#8**	Search (supplement* OR therapy[MeSH Terms] OR patients[MeSH Terms] OR patient*[Title/Abstract] OR therap* OR cross-sectional[Title/Abstract] OR trial*[Title/Abstract] OR pregnan* OR child* OR adolescent* OR infant* OR gene* OR mendelian OR prevalenc* OR protocol[Title/Abstract]), 14553783
**#7**	#6 AND #4, 1955
**#6**	#1 AND #2 AND #3, 7575
**#5**	Search (supplement* OR therapy[MeSH Terms] OR patients[MeSH Terms] OR patient*[Title/Abstract] OR therap* OR cross-sectional[Title/Abstract] OR trial*[Title/Abstract] OR pregnan* OR child* OR adolescent* OR gene* OR mendelian OR prevalenc* OR protocol[Title/Abstract]), 14368617
**#4**	Search (cohort OR longitudinal OR prospectiv* OR “nested case-control”), 2356311
**#3**	Search (osteoporosis OR "bone loss" OR "bone mineral density"), 122123
**#2**	Search (aged[MeSH Terms] OR aging[MeSH Terms] OR "Aged, 80 and over"[Mesh] OR "old age people" OR elder* OR adult* OR retired OR ancient*), 6919264
**#1**	Search (vitamin d[MeSH Terms] OR vitamin d deficiency[MeSH Terms] OR 25-hydroxyvitamin d[MeSH Terms] OR “vitamin d”[Title/Abstract] OR “25-hydroxyvitamin d”[Title/Abstract] OR “25(OH)D” [Title/Abstract] OR “1,25(OH)2D”[Title/Abstract] OR “1,25 dihydroxyvitamin d”[Title/Abstract] OR 1,25 dihydroxyvitamin d[MeSH Terms]), 88545

8) **Search 8 (physical functionality)**
Database PubMed
Date 29th March 2019

Total records 122
String:

((((((vitamin d[MeSH Terms] OR vitamin d deficiency[MeSH Terms] OR 25-hydroxyvitamin d[MeSH Terms] OR “vitamin d”[Title/Abstract] OR “25-hydroxyvitamin d”[Title/Abstract] OR “25(OH)D” [Title/Abstract] OR “1,25(OH)2D”[Title/Abstract] OR “1,25 dihydroxyvitamin d”[Title/Abstract] OR 1,25 dihydroxyvitamin d[MeSH Terms]))) AND ((aged[MeSH Terms] OR aging[MeSH Terms] OR "Aged, 80 and over"[Mesh] OR "old age people" OR elder* OR adult* OR retired OR ancient*))) AND (("physical performance" OR "physical function*" OR "ADL" OR "IADL" OR fall* OR fractur* OR balance))) AND ((cohort OR longitudinal OR prospectiv* OR “nested case-control”))) NOT ((supplement* OR therapy[MeSH Terms] OR patients[MeSH Terms] OR patient*[Title/Abstract] OR therap* OR cross-sectional[Title/Abstract] OR trial*[Title/Abstract] OR pregnan* OR child* OR adolescent* OR gene* OR mendelian OR prevalenc* OR protocol[Title/Abstract])) Filters: Publication date from 2000/01/01 to 2019/03/01; Humans

**#10**	**#9 Filters: Publication date from 2000/01/01 to 2019/03/01, Humans, 122**
**#9**	(#4 AND #8) NOT #5, 145
**#8**	Search (cohort OR longitudinal OR prospectiv* OR “nested case-control”), 2356311
**#7**	#4 NOT #5 Filters: Publication date from 2000/01/01 to 2019/03/01, Humans, 311
**#6**	#4 NOT #5 Filters: Publication date from 2000/01/01 to 2019/03/01, 339
**#5**	Search (supplement* OR therapy[MeSH Terms] OR patients[MeSH Terms] OR patient*[Title/Abstract] OR therap* OR cross-sectional[Title/Abstract] OR trial*[Title/Abstract] OR pregnan* OR child* OR adolescent* OR gene* OR mendelian OR prevalenc* OR protocol[Title/Abstract]), 14368617
**#4**	#1 AND #2 AND #3, 5334
**#3**	Search ("physical performance" OR "physical function*" OR "ADL" OR "IADL" OR fall* OR fractur*), 536420
**#2**	Search (aged[MeSH Terms] OR aging[MeSH Terms] OR "Aged, 80 and over"[Mesh] OR "old age people" OR elder* OR adult* OR retired OR ancient*), 6919264
**#1**	Search (vitamin d[MeSH Terms] OR vitamin d deficiency[MeSH Terms] OR 25-hydroxyvitamin d[MeSH Terms] OR “vitamin d”[Title/Abstract] OR “25-hydroxyvitamin d”[Title/Abstract] OR “25(OH)D” [Title/Abstract] OR “1,25(OH)2D”[Title/Abstract] OR “1,25 dihydroxyvitamin d”[Title/Abstract] OR 1,25 dihydroxyvitamin d[MeSH Terms]), 88545

9) **Search 9 (cognitive functionality)**
Database PubMed
Date 28th March 2019
Total records 31
String:

((((((vitamin d[MeSH Terms] OR vitamin d deficiency[MeSH Terms] OR 25-hydroxyvitamin d[MeSH Terms] OR “vitamin d”[Title/Abstract] OR “25-hydroxyvitamin d”[Title/Abstract] OR “25(OH)D” [Title/Abstract] OR “1,25(OH)2D”[Title/Abstract] OR “1,25 dihydroxyvitamin d”[Title/Abstract] OR 1,25 dihydroxyvitamin d[MeSH Terms]))) AND ((aged[MeSH Terms] OR aging[MeSH Terms] OR "Aged, 80 and over"[Mesh] OR "old age people" OR elder* OR adult* OR retired OR ancient*))) AND (("cognitive function*" OR "cognitive declin*" OR "cognitive performanc*" OR "cognitive impairment*" OR "dementia" OR "Alzheimer"))) AND ((cohort OR longitudinal OR prospectiv* OR “nested case-control”))) NOT ((supplement* OR therapy[MeSH Terms] OR patients[MeSH Terms] OR patient*[Title/Abstract] OR therap* OR cross-sectional[Title/Abstract] OR trial*[Title/Abstract] OR pregnan* OR child* OR adolescent* OR infant* OR gene* OR mendelian OR prevalenc* OR protocol[Title/Abstract])) Filters: Publication date from 2000/01/01 to 2019/03/01; Humans

**#7**	**#6 Filters: Publication date from 2000/01/01 to 2019/03/01, Humans, 31**
**#6**	(#1 AND #2 AND #3 AND #4) NOT #5, 35

**#5**	Search (supplement* OR therapy[MeSH Terms] OR patients[MeSH Terms] OR patient*[Title/Abstract] OR therap* OR cross-sectional[Title/Abstract] OR trial*[Title/Abstract] OR pregnan* OR child* OR adolescent* OR infant* OR gene* OR mendelian OR prevalenc* OR protocol[Title/Abstract]), 14553783
**#4**	Search (cohort OR longitudinal OR prospectiv* OR "nested case-control"), 2356311
**#3**	Search ("cognitive function*" OR "cognitive declin*" OR "cognitive performanc*" OR "cognitive impairment*" OR "dementia" OR "Alzheimer"), 302300
**#2**	Search (aged[MeSH Terms] OR aging[MeSH Terms] OR "Aged, 80 and over"[Mesh] OR "old age people" OR elder* OR adult* OR retired OR ancient*), 6919264
**#1**	Search (vitamin d[MeSH Terms] OR vitamin d deficiency[MeSH Terms] OR 25-hydroxyvitamin d[MeSH Terms] OR "vitamin d" [Title/Abstract] OR "25-hydroxyvitamin d" [Title/Abstract] OR "25(OH)D" [Title/Abstract] OR "1,25(OH)2D" [Title/Abstract] OR "1,25 dihydroxyvitamin d" [Title/Abstract] OR 1,25 dihydroxyvitamin d[MeSH Terms]), 88545

10) **Search 10 (healthy years)**
Database PubMed
Date 28th March 2019
Total records 27
String:

((((((vitamin d[MeSH Terms] OR vitamin d deficiency[MeSH Terms] OR 25-hydroxyvitamin d[MeSH Terms] OR "vitamin d"[Title/Abstract] OR "25-hydroxyvitamin d"[Title/Abstract] OR "25(OH)D" [Title/Abstract] OR "1,25(OH)2D"[Title/Abstract] OR "1,25 dihydroxyvitamin d"[Title/Abstract] OR 1,25 dihydroxyvitamin d[MeSH Terms]))) AND ((aged[MeSH Terms] OR aging[MeSH Terms] OR "Aged, 80 and over"[Mesh] OR "old age people" OR elder* OR adult* OR retired OR ancient*))) AND (("healthy ageing" OR "healthy aging" OR "health status" OR "wellbeing" OR "well-being" OR "quality of life" OR "healthy year*" OR "illness free" OR DALY OR "disability-adjusted life year*" OR "years of life lost" OR "YLL" OR "year* of potential life lost" OR YPLL OR "potential years of life lost" OR PYLL))) AND ((cohort OR longitudinal OR prospectiv* OR "nested case-control"))) NOT ((supplement* OR therapy[MeSH Terms] OR patients[MeSH Terms] OR patient*[Title/Abstract] OR therap* OR cross-sectional[Title/Abstract] OR trial*[Title/Abstract] OR pregnan* OR child* OR adolescent* OR infant* OR gene* OR mendelian OR prevalenc* OR protocol[Title/Abstract])) Filters: Publication date from 2000/01/01 to 2019/03/01; Humans

**#7**	**#6 Filters: Publication date from 2000/01/01 to 2019/03/01, Humans, 27**
**#6**	(#1 AND #2 AND #3 AND #4) NOT #5, 30
**#5**	Search (supplement* OR therapy[MeSH Terms] OR patients[MeSH Terms] OR patient*[Title/Abstract] OR therap* OR cross-sectional[Title/Abstract] OR trial*[Title/Abstract] OR pregnan* OR child* OR adolescent* OR infant* OR gene* OR mendelian OR prevalenc* OR protocol[Title/Abstract]), 14553783
**#4**	Search (cohort OR longitudinal OR prospectiv* OR "nested case-control"), 2356311
**#3**	Search ("healthy ageing" OR "healthy aging" OR "health status" OR "wellbeing" OR "well-being" OR "quality of life" OR "healthy year*" OR "illness free" OR DALY OR "disability-adjusted life year*" OR "years of life lost" OR "YLL" OR "year* of potential life lost" OR YPLL OR "potential years of life lost" OR PYLL), 491735
**#2**	Search (aged[MeSH Terms] OR aging[MeSH Terms] OR "Aged, 80 and over"[Mesh] OR "old age people" OR elder* OR adult* OR retired OR ancient*), 6919264
**#1**	Search (vitamin d[MeSH Terms] OR vitamin d deficiency[MeSH Terms] OR 25-hydroxyvitamin d[MeSH Terms] OR "vitamin d" [Title/Abstract] OR "25-hydroxyvitamin d" [Title/Abstract] OR "25(OH)D" [Title/Abstract] OR "1,25(OH)2D" [Title/Abstract] OR "1,25 dihydroxyvitamin d" [Title/Abstract] OR 1,25 dihydroxyvitamin d[MeSH Terms), 88545

11) **Search 11**
Database Scopus
Date 5th March 2019
Total records 209
String:

ALL ("vitamin d") OR ALL ("25-hydroxyvitamin d") OR ALL ("25(OH)D") OR ALL ("1,25 dihydroxyvitamin d") OR ALL ("1,25(OH)2D") AND (ALL ("aging") OR ALL ("ageing") OR ALL ("elder*") OR ALL ("80 and over") OR ALL ("50 and over") OR ALL ("adult*")) AND (ALL ("healthy ageing") OR ALL ("health status") OR ALL ("mortality") OR ALL ("ill health") OR ALL ("death") OR ALL ("wellbeing") OR ALL ("well-being") OR ALL ("diseas*") OR ALL ("physical function") OR ALL ("cognitive function") OR ALL ("quality of life") OR ALL ("physical performance") OR ALL ("frail*") OR ALL ("longevity")) AND NOT ALL ("patient*") AND NOT ALL ("therap*") AND NOT ALL ("treat*") AND NOT ALL (human) AND (LIMIT-TO (SRCTYPE , "j")) AND (LIMIT-TO (DOCTYPE , "ar")) AND (LIMIT-TO (PUBYEAR , 2019) OR LIMIT-TO (PUBYEAR , 2018) OR LIMIT-TO (PUBYEAR , 2017) OR LIMIT-TO (PUBYEAR , 2016) OR LIMIT-TO (PUBYEAR , 2015) OR LIMIT-TO (PUBYEAR , 2014) OR LIMIT-TO (PUBYEAR , 2013) OR LIMIT-TO (PUBYEAR , 2012) OR LIMIT-TO (PUBYEAR , 2011) OR LIMIT-TO (PUBYEAR , 2010) OR LIMIT-TO (PUBYEAR , 2009) OR LIMIT-TO (PUBYEAR , 2008) OR LIMIT-TO (PUBYEAR , 2007) OR LIMIT-TO (PUBYEAR , 2006) OR LIMIT-TO (PUBYEAR , 2005) OR LIMIT-TO (PUBYEAR , 2004) OR LIMIT-TO (PUBYEAR , 2003) OR LIMIT-TO (PUBYEAR , 2002) OR LIMIT-TO (PUBYEAR , 2001) OR LIMIT-TO (PUBYEAR , 2000)) AND (EXCLUDE (EXACTKEYWORD , "Nonhuman") OR EXCLUDE (EXACTKEYWORD , "Animal") OR EXCLUDE (EXACTKEYWORD , "Animal Tissue") OR EXCLUDE (EXACTKEYWORD , "Animal Experiment")) AND (LIMIT-TO (LANGUAGE , "English") OR LIMIT-TO (LANGUAGE , "Spanish") OR LIMIT-TO (LANGUAGE , "Italian"))

12) Search 12

Database Web of Science
Date 6th March 2019
Total records 377
String:

**# 16**	**377**	**#5 NOT #14**<br>**Indexes=SCI-EXPANDED, SSCI, A&HCI, CPCI-S, CPCI-SSH, ESCI Timespan=2000-2019**
# 15	385	#5 NOT #14<br>Indexes=SCI-EXPANDED, SSCI, A&HCI, CPCI-S, CPCI-SSH, ESCI Timespan=All years
# 14	15,234,767	#6 OR #7 OR #8 OR #11 OR #12 OR #13<br>Indexes=SCI-EXPANDED, SSCI, A&HCI, CPCI-S, CPCI-SSH, ESCI Timespan=All years
# 13	1,028,730	TS=(animal*)<br>Indexes=SCI-EXPANDED, SSCI, A&HCI, CPCI-S, CPCI-SSH, ESCI Timespan=All years
# 12	1,013,582	TI=(trial OR meta-analys* OR review* OR cross-sectional OR protocol*)<br>Indexes=SCI-EXPANDED, SSCI, A&HCI, CPCI-S, CPCI-SSH, ESCI Timespan=All years
# 11	12,559,436	ALL=(therap* OR treat* OR drug* OR patient* OR pharmac* OR operativ* OR postoperativ* OR surger* OR hospital* OR inpatient*)<br>Indexes=SCI-EXPANDED, SSCI, A&HCI, CPCI-S, CPCI-SSH, ESCI Timespan=All years
# 10	4,314,428	TI=(therap* OR treat* OR drug* OR patient* OR pharmac* OR operativ* OR postoperativ* OR surger* OR hospital* OR inpatient*)<br>Indexes=SCI-EXPANDED, SSCI, A&HCI, CPCI-S, CPCI-SSH, ESCI Timespan=All years
# 9	919,036	TI=(trial OR meta-analys* OR review* OR cross-sectional)<br>Indexes=SCI-EXPANDED, SSCI, A&HCI, CPCI-S, CPCI-SSH, ESCI Timespan=All years
# 8	1,134,488	TI=(gene OR genet* OR polymorph*)<br>Indexes=SCI-EXPANDED, SSCI, A&HCI, CPCI-S, CPCI-SSH, ESCI Timespan=All years
# 7	328,321	TI=(supplement* OR diet* OR intak*)<br>Indexes=SCI-EXPANDED, SSCI, A&HCI, CPCI-S, CPCI-SSH, ESCI Timespan=All years
# 6	1,402,603	TI=(child* OR infant* OR adolescent* OR young OR youth OR gestational OR fetal* OR pregnan* OR neonatal* OR maternal*)<br>Indexes=SCI-EXPANDED, SSCI, A&HCI, CPCI-S, CPCI-SSH, ESCI Timespan=All years
# 5	1,535	#1 AND #2 AND #3 AND #4<br>Indexes=SCI-EXPANDED, SSCI, A&HCI, CPCI-S, CPCI-SSH, ESCI Timespan=All years

**# 4**	1,417,741	ALL=(cohort* OR longitudinal OR prospectiv*)<br>Indexes=SCI-EXPANDED, SSCI, A&HCI, CPCI-S, CPCI-SSH, ESCI Timespan=All years
**# 3**	4,457,786	ALL=(healthy ageing OR healthy aging OR health status OR quality of life OR mortality OR death* OR longevity OR survival OR ill health OR cognitive function* OR physical function* OR physical performance* OR frail*)<br>Indexes=SCI-EXPANDED, SSCI, A&HCI, CPCI-S, CPCI-SSH, ESCI Timespan=All years
**# 2**	3,382,836	ALL=(aging OR ageing OR 80 and over OR 50 and over OR elder* OR ancient*)<br>Indexes=SCI-EXPANDED, SSCI, A&HCI, CPCI-S, CPCI-SSH, ESCI Timespan=All years
**# 1**	43,429	TI=(vitamin d OR 25-hydroxyvitamin d OR 25(OH)D OR 1,25 dihydroxyvitamin d OR 1,25(OH)2D)<br>Indexes=SCI-EXPANDED, SSCI, A&HCI, CPCI-S, CPCI-SSH, ESCI Timespan=All years
