# Supplementary material for: Vitamin D as a Biomarker of Ill-Health among the Over-50s: A Systematic Review of Cohort Studies
Source: Nutrients. 2019 Oct 6;11(10):2384. doi: 10.3390/nu11102384 (PMC6836048; doi:10.3390/nu11102384)
Supplement: Supplementary file 1 [file nutrients-11-02384-s001.zip › Table S2 Studies included.pdf]

Table S2: Studies included

Cohorts and references of studies included in this Systematic Review

Cohorte Lausannoise (CoLaus) study

Marques-Vidal 2015

Marques-Vidal P, Vollenweider P, Guessous I, et al. Serum Vitamin D Concentrations Are Not Associated with Insulin Resistance in Swiss Adults. J Nutr. 2015;145(9):2117-2122

Concord Health and Aging in Men Project (CHAMP)

Hirani 2018

Hirani V, Cumming RG, Naganathan V, et al. Longitudinal Associations Between Vitamin D Metabolites and Sarcopenia in Older Australian men: The Concord Health and Aging in Men Project. JOURNALS Gerontol Ser A-BIOLOGICAL Sci Med Sci. 2018;73(1, SI):131-138

Copenhagen City Heart Study

Afzal 2014

Afzal S, Lange P, Bojesen SE, Freiberg JJ, Nordestgaard BG. Plasma 25-hydroxyvitamin D, lung function and risk of chronic obstructive pulmonary disease. Thorax. 2014;69(1):24-31

Afzal 2013

Afzal S, Nordestgaard BG, Bojesen SE. Plasma 25-hydroxyvitamin D and risk of non-melanoma and melanoma skin cancer: a prospective cohort study. J Invest Dermatol. 2013;133(3):629-636

Brøndum-Jacobsen 2012

Brøndum-Jacobsen P, Benn M, Jensen GB, Nordestgaard BG. 25-Hydroxyvitamin D Levels and Risk of Ischemic Heart Disease, Myocardial Infarction, and Early Death. Arterioscler Thromb Vasc Biol. 2012;32(11):2794-2802

Copenhagen General Population Study

Afzal 2014

Afzal S, Lange P, Bojesen SE, Freiberg JJ, Nordestgaard BG. Plasma 25-hydroxyvitamin D, lung function and risk of chronic obstructive pulmonary disease. Thorax. 2014;69(1):24-31

EPIC cohort

Khaw 2014

Khaw K-T, Lubetz R, Wareham N. Serum 25-hydroxyvitamin D, mortality, and incident cardiovascular disease, respiratory disease, cancers, and fractures: a 13-y prospective population study. Am J Clin Nutr. 2014;100(5):1361-1370

Health Professionals Follow-up Study

Platz 2004

Platz EA, Leitzmann MF, Hollis BW, Willett WC, Giovannucci E. Plasma 1,25-dihydroxy- and 25-hydroxyvitamin D and subsequent risk of prostate cancer. Cancer Causes Control. 2004;15(3):255-265

Hisayama study

Umehara 2017

Umehara K, Mukai N, Hata J, et al. Association Between Serum Vitamin D and All-Cause and Cause-Specific Death in a General Japanese Population - The Hisayama Study -. Circ J. 2017;81(9):1315+

Kuopio Ischaemic Heart Disease Risk Factor Study (KIHD)

Aregbesola 2013

Aregbesola A, Voutilainen S, Nurmi T, Virtanen JK, Ronkainen K, Tuomainen T. Serum 25-hydroxyvitamin D 3 and the risk of pneumonia in an ageing general population. J Epidemiol Community Health. 2013;67(6):533-536

Mursu 2015

Mursu J, Nurmi T, Voutilainen S, Tuomainen T-P, Virtanen JK. The association between serum 25-hydroxyvitamin D3 concentration and risk of disease death in men: modification by magnesium intake. Eur J Epidemiol. 2015;30(4):343-347

Lebanese cohort

Arabi 2012

Arabi A, Baddoura R, El-Rassi R, El-Hajj Fuleihan G. PTH level but not 25 (OH) vitamin D level predicts bone loss rates in the elderly. Osteoporos Int. 2012;23(3):971-980

Melbourne Collaborative Cohort Study (MCCS)

Heath 2017

Heath AK, Williamson EJ, Kvaskoff D, et al. 25-Hydroxyvitamin D concentration and all-cause mortality: the Melbourne Collaborative Cohort Study. Public Health Nutr. 2017;20(10):1775-1784

Osteoporotic Fractures in Men Study (MrOS)

Barrett-Connor 2012

Barrett-Connor E, Laughlin GA, Li H, et al. The association of concurrent vitamin D and sex hormone deficiency with bone loss and fracture risk in older men: The osteoporotic fractures in men (MrOS) study. J Bone Miner Res. 2012;27(11):2306-2313

Cauley 2010

Cauley JA, Parimi N, Ensrud KE, et al. Serum 25-hydroxyvitamin D and the risk of hip and nonspine fractures in older men. J Bone Miner Res. 2010;25(3):545-553

Swanson 2015

Swanson CM, Srikanth P, Lee CG, et al. Associations of 25-Hydroxyvitamin D and 1,25-Dihydroxyvitamin D With Bone Mineral Density, Bone Mineral Density Change, and Incident Nonvertebral Fracture. J Bone Miner Res. 2015;30(8):1403-1413

Otasha-Kenshin study

Shimizu 2015

Shimizu Y, Kim H, Yoshida H, Shimada H, Suzuki T. Serum 25-hydroxyvitamin D level and risk of falls in Japanese community-dwelling elderly women: a 1-year follow-up study. Osteoporos Int. 2015;26(8):2185-2192

REGICOR (Registre GIronı´ del COR) population cohort study

Vàzquez-Oliva 2018

Vázquez-Oliva G, Zamora A, Ramos R, et al. Analysis of Plasma Albumin, Vitamin D, and Apolipoproteins A and B as Predictive Coronary Risk Biomarkers in the REGICOR Study. Rev Española Cardiol (English Ed. 2018;71(11):910-916

Swedish farmers study

Holmberg 2017

Holmberg S, Rignell-Hydbom A, Lindh CH, Jonsson BAG, Thelin A, Rylander L. High levels of vitamin D associated with less ischemic heart disease - a nested case-control study among rural men in Sweden. Ann Agric Environ Med. 2017;24(2):288-293

The Rancho Bernardo Study

Jassal 2010

Jassal SK, Chonchol M, von Mühlen D, Smits G, Barrett-Connor E. Vitamin D, Parathyroid Hormone, and Cardiovascular Mortality in Older Adults: The Rancho Bernardo Study. Am J Med. 2010;123(12):1114-1120

The Rotterdam Study (RS-I-3) & The Rotterdam Study (RS-II-1)

Licher 2017

Licher S, de Bruijn RFAG, Wolters FJ, Zillikens MC, Ikram MA, Ikram MK. Vitamin D and the Risk of Dementia: The Rotterdam Study. J Alzheimers Dis. 2017;60(3):989-997

Third National Health and Nutrition Examination Survey (NHANES III)

Al-khalidi 2019

Al-khalidi B, Kuk JL, Ardern CI. Journal of Steroid Biochemistry and Molecular Biology Lifetime risk of cardiometabolic mortality according to vitamin D status of middle and older-aged adults : NHANES III mortality follow-up. J Steroid Biochem Mol Biol. 2019;186(August 2018):34-41

Liu 2012

Liu L, Chen M, Hankins SR, et al. Serum 25-hydroxyvitamin D concentration and mortality from heart failure and cardiovascular disease, and premature mortality from all-cause in United States adults. Am J Cardiol. 2012;110(6):834-839

Third National Health and Nutrition Examination Survey (NHANES III) & NHANES 2000-2004

Looker 2013

Looker AC. Serum 25-hydroxyvitamin D and risk of major osteoporotic fractures in older U.S. adults. J Bone Miner Res. 2013;28(5):997-1006

Uppsala Longitudinal Study of Adult men

Olsson 2017

Olsson E, Byberg L, Karlström B, et al. Vitamin D is not associated with incident dementia or cognitive impairment: an 18-y follow-up study in community-living old men. Am J Clin Nutr. 2017;105(4):936-943
