# Supplementary material for: Vitamin D as a Biomarker of Ill-Health among the Over-50s: A Systematic Review of Cohort Studies
Source: Nutrients. 2019 Oct 6;11(10):2384. doi: 10.3390/nu11102384 (PMC6836048; doi:10.3390/nu11102384)
Supplement: Supplementary file 1 [file nutrients-11-02384-s001.zip › Table S3 Characteristics of studies included.pdf]

Table S3: Characteristics of studies included

**Author**	**Afzal 2014**
**Design**	cohort
**Cohort name**	Copenhagen City Heart Study & Copenhagen General Population Study
**Number of cohorts**	2
**Country**	Denmark
**Enrollment year**	*Copenhagen City Heart Study* 1976-1978<br>*Copenhagen General Population Study* 2003
**Baseline year**	*Copenhagen City Heart Study* 1981–1983<br>*Copenhagen General Population Study* 2004-2005
**No. Enrolled**<br>**Total particpants included (% females)**	*Copenhagen City Heart Study*<br>No. of enrolled 10116<br>Total included 10116 (F 56,2%)<br>*Copenhagen General Population Study*<br>No. of enrolled 8391<br>Total included 8391 (F 53%)
**Mean age (range / SD)**	*Copenhagen City Heart Study*<br>Age 58 (47 - 64)<br>*Copenhagen General Population Study*<br>Age 56 (46 - 66)
**Follow-up (years)**	*Copenhagen City Heart Study*<br>20 years from baseline<br>*Copenhagen General Population Study*<br>5 years from baseline

**Author**	**Afzal 2013**
**Design**	cohort
**Cohort name**	Copenhagen City Heart Study
**Number of cohorts**	1

**Country**	Denmark
**Enrollment year**	1981–1983
**Baseline year**	1981–1983
**No. Enrolled**<br>**Total particpants included (% females)**	No. of enrolled 10060<br>Total included 10060 (F 56,2%)
**Mean age (range / SD)**	Age 58 (48 - 65)
**Follow-up (years)**	20 years from baseline

**Author**	**Al-khalidi 2019**
**Design**	cohort
**Cohort name**	Third National Health and Nutrition Examination Survey (NHANES III)
**Number of cohorts**	1
**Country**	USA
**Enrollment year**	1988-1994
**Baseline year**	1988–1994
**No. Enrolled**<br>**Total particpants included (% females)**	No. of enrolled 33994<br>Total included 6896 (F n.a.)
**Mean age (range / SD)**	Age 61.1 (13.9)
**Follow-up (years)**	17.9 years from baseline

**Author**	**Arabi 2012**
**Design**	cohort
**Cohort name**	Lebanese cohort
**Number of cohorts**	1
**Country**	Lebanon
**Enrollment year**	2002-2003
**Baseline year**	2002-2003
**No. Enrolled**<br>**Total particpants included (% females)**	No. of enrolled 195<br>Total included 192 (F 66,7%)
**Mean age (range / SD)**	Age 72.3 (5.1)

**Follow-up (years)**	4 years from baseline

**Author**	**Aregbesola 2013**
**Design**	cohort
**Cohort name**	Kuopio Ischaemic Heart Disease Risk Factor Study (KIHD)
**Number of cohorts**	2
**Country**	Finland
**Enrollment year**	1516 men aged 42, 48, 54 or 60 years enrolled in 1986–1989 + random sample of 920 postmenopausal women aged 53–73 years fro the same area
**Baseline year**	1998-2001
**No. Enrolled**<br>**Total particpants included (% females)**	No. of enrolled 2436<br>Total included 1421 (F 49,1%)
**Mean age (range / SD)**	Age 62.5 (53-73)
**Follow-up (years)**	9.8 years from baseline

**Author**	**Barrett-Connor 2012**
**Design**	cohort
**Cohort name**	Osteoporotic Fractures in Men Study (MrOS)
**Number of cohorts**	1
**Country**	USA
**Enrollment year**	2000-2002
**Baseline year**	2000-2002
**No. Enrolled**<br>**Total particpants included (% females)**	No. of enrolled 5994<br>Total included 1468 (F 0%)
**Mean age (range / SD)**	Age 74 (5.7)
**Follow-up (years)**	4.7 years from baseline

**Author**	**Brøndum-Jacobsen 2012**
**Design**	cohort

**Cohort name**	Copenhagen City Heart Study
**Number of cohorts**	1
**Country**	Denmark
**Enrollment year**	1976-1978
**Baseline year**	1981–1983
**No. Enrolled**<br>**Total particpants included (% females)**	No. of enrolled 12698<br>Total included 10170 (F 56%)
**Mean age (range / SD)**	Age 57 (49-66)
**Follow-up (years)**	21 years from baseline

**Author**	**Cauley 2010**
**Design**	case-cohort
**Cohort name**	Osteoporotic Fractures in Men Study (MrOS)
**Number of cohorts**	1
**Country**	USA
**Enrollment year**	2000-2002
**Baseline year**	2000-2002
**No. Enrolled**<br>**Total particpants included (% females)**	No. of enrolled 5995<br>Total included 1606 (F 0%)<br>Total sample for the non-spine fracture analysis (1929 men, 1494 subcohort and 435 fracture cases)<br>Total sample for hip fracture analysis (1665 men, 1584 subcohort and 81 hip fracture cases)
**Mean age (range / SD)**	Age 73.8 (5.9)
**Follow-up (years)**	5.3 years from baseline

**Author**	**Heath 2017**
**Design**	case-cohort
**Cohort name**	Melbourne Collaborative Cohort Study (MCCS)
**Number of cohorts**	1
**Country**	Australia
**Enrollment year**	1990-1994

**Baseline year**	1990-1994
**No. Enrolled**<br>**Total particpants included (% females)**	No. of enrolled 41514<br>Total included 4964 (F 46,5%)<br>Cases 2410<br>Sub-cohort of controls 2996
**Mean age (range / SD)**	Age 54.5 (40-69)
**Follow-up (years)**	13.7 years from baseline

**Author**	**Hirani 2018**
**Design**	cohort
**Cohort name**	Concord Health and Aging in Men Project (CHAMP)
**Number of cohorts**	1
**Country**	Australia
**Enrollment year**	2005-2007
**Baseline year**	2005-2007
**No. Enrolled**<br>**Total particpants included (% females)**	No. of enrolled 1705<br>Total included 1705 (F 0%)
**Mean age (range / SD)**	Age 77.8 (4.6)
**Follow-up (years)**	5 years from baseline

**Author**	**Holmberg 2017**
**Design**	nested case-control
**Cohort name**	Swedish farmers study
**Number of cohorts**	1
**Country**	Sweden
**Enrollment year**	1989
**Baseline year**	1990-1991
**No. Enrolled**<br>**Total particpants included (% females)**	No. of enrolled 2350<br>Total included 529 (F 0%)<br>Cases 276

	Controls 276
**Mean age (range / SD)**	Age 51.5 (n.a.)
**Follow-up (years)**	20 years from baseline

**Author**	**Jassal 2010**
**Design**	cohort
**Cohort name**	The Rancho Bernardo Study
**Number of cohorts**	1
**Country**	USA
**Enrollment year**	1972
**Baseline year**	1997-1999
**No. Enrolled**<br>**Total particpants included (% females)**	No. of enrolled 2062<br>Total included 1073 (F 61,7%)
**Mean age (range / SD)**	Age 74 (10)
**Follow-up (years)**	6.8 years from baseline

**Author**	**Khaw 2014**
**Design**	cohort
**Cohort name**	EPIC cohort
**Number of cohorts**	1
**Country**	UK
**Enrollment year**	1993-1997
**Baseline year**	1997-2000
**No. Enrolled**<br>**Total particpants included (% females)**	No. of enrolled 25639<br>Total included 14641 (F 55,7%)
**Mean age (range / SD)**	Age 62.9 (9)
**Follow-up (years)**	13 years from baseline

**Author**	**Licher 2017**

**Design**	cohort
**Cohort name**	The Rotterdam Study (RS-I-3) & The Rotterdam Study (RS-II-1)
**Number of cohorts**	2
**Country**	Netherland
**Enrollment year**	*The Rotterdam Study (RS-I-3)* 1990<br>*The Rotterdam Study (RS-II-1)* 2000
**Baseline year**	*The Rotterdam Study (RS-I-3)* 1997-1999<br>*The Rotterdam Study (RS-II-1)* 2000-2001
**No. Enrolled**<br>**Total particpants included (% females)**	*The Rotterdam Study (RS-I-3)*<br>No. of enrolled 7983<br>Total included 3043,5 (F 56,9%)<br>*The Rotterdam Study (RS-II-1)*<br>No. of enrolled 3011<br>Total included 3043,5 (F 56,9%)
**Mean age (range / SD)**	Age 69.2 (8.2)
**Follow-up (years)**	15 years from baseline

**Author**	**Liu 2012**
**Design**	cohort
**Cohort name**	Third National Health and Nutrition Examination Survey (NHANES III)
**Number of cohorts**	1
**Country**	USA
**Enrollment year**	1988-1994
**Baseline year**	1988-1994
**No. Enrolled**<br>**Total particpants included (% females)**	No. of enrolled n.a<br>Total included 13131 (F 53,3%)
**Mean age (range / SD)**	Age 55 (n.a.)
**Follow-up (years)**	8 years from baseline

**Author**	**Looker 2013**

**Design**	cohort
**Cohort name**	NHANES III & NHANES 2000-2004
**Number of cohorts**	2
**Country**	USA
**Enrollment year**	*NHANES III* 1988- 1994<br>*NHANES 2000-2004* 2000-2004
**Baseline year**	*NHANES 2000-2004* 2000-2004
**No. Enrolled**<br>**Total particpants included (% females)**	*NHANES III*<br>No. of enrolled 8303<br>Total included 2946 (F 78,6%)<br>*NHANES 2000-2004*<br>No. of enrolled 4189<br>Total included 1803 (F 128,5%)
**Mean age (range / SD)**	Age 74 (n.a.)
**Follow-up (years)**	7 years from baseline

**Author**	**Marques-Vidal 2015**
**Design**	cohort
**Cohort name**	Cohorte Lausannoise (CoLaus) study
**Number of cohorts**	1
**Country**	Switzerland
**Enrollment year**	2003-2006
**Baseline year**	2003-2006
**No. Enrolled**<br>**Total particpants included (% females)**	No. of enrolled 6733<br>Total included 3841 (F 57,6%)
**Mean age (range / SD)**	Age 52.2 (10.5)
**Follow-up (years)**	5.5 years from baseline

**Author**	**Mursu 2015**
**Design**	cohort

**Cohort name**	Kuopio Ischaemic Heart Disease Risk Factor Study (KIHD)
**Number of cohorts**	1
**Country**	Finland
**Enrollment year**	1984-1989
**Baseline year**	1984-1989
**No. Enrolled**<br>**Total particpants included (% females)**	No. of enrolled 2682<br>Total included 1892 (F 0%)
**Mean age (range / SD)**	Age (42- 60)
**Follow-up (years)**	26 years from baseline

**Author**	**Olsson 2017**
**Design**	cohort
**Cohort name**	Uppsala Longitudinal Study of Adult men
**Number of cohorts**	1
**Country**	Sweden
**Enrollment year**	1970
**Baseline year**	1991-1995
**No. Enrolled**<br>**Total particpants included (% females)**	No. of enrolled 2322<br>Total included 1182 (F 0%)
**Mean age (range / SD)**	Age 71 (69- 74)
**Follow-up (years)**	18 years from baseline

**Author**	**Platz 2004**
**Design**	nested case-control
**Cohort name**	Health Professionals Follow-up Study
**Number of cohorts**	1
**Country**	USA
**Enrollment year**	1986
**Baseline year**	1993-1995
**No. Enrolled**	No. of enrolled 51529

**Total particpants included (% females)**	Total included 920 (F 0%)<br>Cases 460<br>Controls 460
**Mean age (range / SD)**	Age 66.5 (7.4)
**Follow-up (years)**	5 years from baseline

**Author**	**Shimizu 2015**
**Design**	cohort
**Cohort name**	Otasha-Kenshin study
**Number of cohorts**	1
**Country**	Japan
**Enrollment year**	2008
**Baseline year**	2008
**No. Enrolled**<br>**Total particpants included (% females)**	No. of enrolled 1399<br>Total included 1393 (F 100%)
**Mean age (range / SD)**	Age 78.6 (75-90)
**Follow-up (years)**	1 years from baseline

**Author**	**Swanson 2015**
**Design**	cohort
**Cohort name**	Osteoporotic Fractures in Men Study (MrOS)
**Number of cohorts**	1
**Country**	USA
**Enrollment year**	2000-2002
**Baseline year**	2000-2002
**No. Enrolled**<br>**Total particpants included (% females)**	No. of enrolled 5994<br>Total included 1000 (F 0%)
**Mean age (range / SD)**	Age 74.6 (6.2)
**Follow-up (years)**	5.1 years from baseline

**Author**	**Umehara 2017**
**Design**	cohort
**Cohort name**	Hisayama study
**Number of cohorts**	1
**Country**	Japan
**Enrollment year**	1961
**Baseline year**	2002-2003
**No. Enrolled**<br>**Total particpants included (% females)**	No. of enrolled 3328<br>Total included 3292 (F 57,2%)
**Mean age (range / SD)**	Age 62 (12.5)
**Follow-up (years)**	9.5 years from baseline

**Author**	**Vàzquez-Oliva 2018**
**Design**	case-cohort
**Cohort name**	REGICOR (Registre GIronı´ del COR) population cohort study
**Number of cohorts**	1
**Country**	Spain
**Enrollment year**	2005
**Baseline year**	2005
**No. Enrolled**<br>**Total particpants included (% females)**	No. of enrolled 6352<br>Total included 775 (F 50,2%)<br>Cases 105<br>Random sub-cohort controls 667
**Mean age (range / SD)**	Age 53.7 (10.6)
**Follow-up (years)**	5 years from baseline
